# Effect of Tyrosin Kinase Inhibitors on NK Cell and ILC3 Development and Function

**DOI:** 10.3389/fimmu.2018.02433

**Published:** 2018-10-23

**Authors:** Laura Damele, Elisa Montaldo, Lorenzo Moretta, Chiara Vitale, Maria Cristina Mingari

**Affiliations:** ^1^Dipartimento Medicina Sperimentale, Università degli Studi di Genova, Genova, Italy; ^2^Centre of Excellence for Biomedical Research, Università degli Studi di Genova, Genova, Italy; ^3^IRCCS G. Gaslini, Genova, Italy; ^4^Immunology Area Lab, Pediatric Hospital Bambino Gesù, Rome, Italy; ^5^UO Immunologia, IRCCS Ospedale Policlinico San Martino, Genova, Italy

**Keywords:** innate lymphocyte cells, NK cell development, tyrosin kinase inhibitors, CML-chronic myelogenous leukemia, ILC3

## Abstract

Tyrosin kinase inhibitors (TKI) sharply improved the prognosis of Chronic Myeloid Leukemia (CML) and of Philadelphia^+^ Acute Lymphoblastic Leukemia (Ph^+^ALL) patients. However, TKI are not curative because of the development of resistance and lack of complete molecular remission in the majority of patients. Clinical evidences would support the notion that patient's immune system may play a key role in preventing relapses. In particular, increased proportions of terminally differentiated CD56^+^CD16^+^CD57^+^ NK cells have been reported to be associated with successful Imatinib therapy discontinuation or with a deep molecular response in Dasatinib-treated patients. In view of the potential role of NK cells in immune-response against CML, it is important to study whether any TKI have an effect on the NK cell development and identify possible molecular mechanism(s) by which continuous exposure to *in vitro* TKI may influence NK cell development and repertoire. To this end, CD34^+^ hematopoietic stem cells (HSC) were cultured in the absence or in the presence of Imatinib, Nilotinib, or Dasatinib. We show that all compounds exert an inhibitory effect on CD56^+^ cell recovery. In addition, Dasatinib sharply skewed the repertoire of CD56^+^ cell population, leading to an impaired recovery of CD56^+^CD117^−^CD16^+^CD94/NKG2A^+^EOMES^+^ mature cytotoxic NK cells, while the recovery of CD56^+^CD117^+^CD94/NKG2A^−^RORγt^+^ IL-22-producing ILC3 was not affected. This effect appears to involve the Dasatinib–mediated inhibition of Src kinases and, indirectly, of STAT5-signaling activation in CD34^+^ cells during first days of culture. Our studies, reveal a possible mechanism by which Dasatinib may interfere with the proliferation and maturation of fully competent NK cells, i.e., by targeting signaling pathways required for differentiation and survival of NK cells but not of ILC3.

## Introduction

Therapy with tyrosin kinase inhibitors (TKI) has greatly improved the prognosis of Chronic Myeloid Leukemia (CML) and of Philadelphia^+^ Acute Lymphoblastic Leukemia (Ph^+^ALL) patients (1–3). However, despite their efficacy, TKI cannot be considered as curative therapeutic agents, because the majority of patients develop resistance or lack complete molecular remission (4–6). Moreover, patients undergoing life-long treatment may experience adverse effects that compromise their quality of life (6–8). Thus, during the past several years, the achievement of therapy discontinuation, allowing long treatment-free remissions (TFR) became a main area of investigation (8). Recent clinical trials indicated that ~40% of patients with deep molecular response (DMR), who undergo therapy discontinuation, remain free of relapse for several years (8–10). Clinical evidences would support the notion that the patient's immune system may play a key role either by eradicating leukemia or by exerting a successful long-lasting control of residual leukemic cells. Indeed, patients with an efficient effector arm of their immune system display a significantly longer TFR with a DMR (11–13). Thus, ongoing major efforts are aimed to identify immune mechanisms and biomarkers that may help to select patients who are suitable for successful therapy discontinuation upon achievement of a DMR. In this context, analysis of natural killer (NK) cells, capable of a potent anti-leukemia activity, could offer a clue to identify such patients (14).

NK cells represent an important component of the innate immunity. Their function is finely tuned by inhibitory and activating receptors. Remarkably, NK cells have been shown to play an important role in the favorable clinical outcome of patients with high risk leukemias undergoing haploidentical Hematopoietic Stem Cell Transplant (15–18). NK cells can recognize and kill leukemic blasts, particularly those displaying down-regulation of HLA-class I molecules (failing to interact with HLA-specific inhibitory NK receptors such as KIR and CD94/NKG2A), and/or over-expressing ligands recognized by activating NK receptors (including Natural Cytotoxic Receptors, NCR, NKG2D and DNAM-1) (16, 19). Certain activating KIR, present only in some individuals, may also positively contribute to the anti-leukemia activity (14, 20, 21).

NK cell development occurs primarily in the bone marrow (BM), requires the expression of E4BP4 (NFIL3), Tbx21 (T-bet), eomesodermin (EOMES) transcription factors (TF), and proceeds through a multi-step process, characterized by the sequential acquisition of surface receptor markers (including CD161, CD56, CD94/NKG2A, LFA-1, CD16, KIRs, and CD57) and given functional capabilities (22–24). NK cells are developmentally related to Innate Lymphoid Cells (ILCs) as they derive from a common DNA-binding protein inhibitor (ID2)-positive hematopoietic precursor (24). Members of ILC family are thought to play a relevant role in innate defenses against pathogens, in epithelial tissue homeostasis and in lymphoid structure organization. Three main groups of ILCs have been identified: ILC1, ILC2, and ILC3, on the basis of their cytokine profile and transcription factors (TF) required for their differentiation (25). *In vitro* models of human NK cell development from umbilical cord blood (UCB)-derived CD34^+^ cells revealed that these precursors can give rise both to NK cells and ILC3. The expression of CD94/NKG2A and LFA-1 marks CD161^+^CD56^+^CD117^−^CD7^+^ NK cells that express NCR, cytolytic granules and production of IFN-γ. On the other hand, the lack of expression of CD94/NKG2A and LFA-1 (CD161^+^CD56^+^CD117^+^CD7^−^LFA-1^−^CD94/NKG2A^−^) identifies a heterogeneous cell subset, that may contain both NK cell precursors and ILC3, characterized by the expression of RAR-related orphan receptor gamma (RORγt) TF and by the ability to produce IL-22 (26, 27).

In the past few years, the effects of TKI on the NK cell repertoire and function have been analyzed in several studies (28). Of note, increased proportions of terminally differentiated cytolytic CD56^+^CD16^+^CD57^+^ NK cells were found in patients that achieved a successful Imatinib therapy discontinuation or in Dasatinib-treated patients with a DMR (12, 29–32). Recently, it has also been suggested that KIR genotype may represent a new biomarker for response to TKI therapy (33–35). On the other hand, previous *in vitro* studies reported conflicting results on the effect of different TKI on NK cell proliferation and function (28).

In view of the potential role of NK cells in the control of CML, it is important to study the effect of TKI not only on mature NK cells, but also on NK cells undergoing maturation. Notably, TKI may impair hematopoiesis, consequent to the inhibitory effect on c-KIT transduction pathway. Moreover, Dasatinib inhibits Src kinase, also involved in the regulation of hematopoiesis. Thus, it is possible that prolonged administration of TKI may affect NK cell differentiation from Hematopoietic Stem Cells (HSC) (24, 36–38). To explore this possibility, whether indeed TKI could influence NK cell development and repertoire, UCB-derived CD34^+^ HSC were cultured in the absence or in the presence of Imatinib, Nilotinib, or Dasatinib. Our results show that all compounds exert an inhibitory effect on cell proliferation. In addition, Dasatinib sharply skewed the repertoire of CD56^+^ cells, with an impaired recovery of CD56^+^CD117^−^CD16^+^CD94/NKG2A^+^EOMES^+^ mature cytotoxic NK cells, paralleled by an enrichment of CD56^+^CD117^+^CD94/NKG2A^−^RORγt^+^ ILC3. This effect appears to involve the Dasatinib–mediated inhibition of Src kinases. Our studies, revealed a mechanism by which Dasatinib may interfere with the maturation of fully competent NK cells, i.e., by targeting signaling pathways required for differentiation of NK cells but not of ILC3.

## Materials and methods

### Cell isolation and *in vitro* culture

Liguria Cord Blood Bank provided UCB samples from healthy individuals. Ethical Committee approved the study and mothers gave their written informed consent according to the Helsinki Declaration. Mononuclear cells were obtained by Ficoll-Lympholyte (Cedarlane, Canada) separation. CD56^−^CD34^+^ cells (>98% purity) were obtained by MACS positive separation (Miltenyi Biotec, Germany). Cells were cultured in RPMI 1640 (Lonza, Belgium) containing 10% human AB serum (Biowest, France), Stem Cell Factor (SCF) (10 ng/ml), Fms-related tyrosine kinase 3 ligand (FLT3-L) (10 ng/ml), Interleukin-7 (IL-7) (20 ng/ml), Interleukin-15 (IL-15) (20 ng/ml), Interleukin-21 (IL-21) (20 ng/ml) (Miltenyi Biotec,), in the absence or in the presence of: Imatinib (IM 5 μM), Nilotinib (NIL 3, 6 μM), Dasatinib (DAS 200 nM) (Selleck Chemicals, USA) at the plasma concentration 30 min post administration, or with Dimethyl sulfoxide (DMSO) at the corresponding concentration of the drugs (D 1:1,000/1:25,000) (Sigma-Aldrich, USA) or with KX2-391 used at different concentrations (Selleck Chemicals). We added TKI, DMSO, or KX2-391 at day 0 and at later intervals i.e., 24 h, 4, 10, or 15 days.

### Monoclonal antibodies (mAbs) and flow cytometry

mAbs were purchased from several companies. A full list of the mAbs utilized is provided in Table 1. All the mAbs were mouse-anti human, with the exception of ROR-γt mAb, Phospho-Stat3 (Tyr705)(D3A7)XP mAb, and Phospho-Stat5 (Tyr694)(D47E7) XP mAb were from Rabbit. To perform cell gating strategy we first identified morphological parameters using FSC-A vs. SSC-A. Then, we performed a further gate in which we analyze the FSC-A vs. FSC-H, in order to limit any interference due to doublets. To assess cell viability we performed analyses with Propidium Iodide and Annexin V.

**Table 1:** List of the mAbs used in the experiments.

**Antigen**	**Antibody clone**	**Fluorochrome**	**Supplier**
CD56	N901	PeCy7	Beckman-Coulter
CD159a	Z199	APC	Beckman-Coulter
CD159a	Z199	PE	Beckman-Coulter
CD158a	EB6B	APC	Beckman-Coulter
CD158b1,b2	GL183	APC	Beckman-Coulter
CD158e1,e2	Z27.3.7	APC	Beckman-Coulter
CD158a	EB6B	PE	Beckman-Coulter
CD158b1,b2	GL183	PE	Beckman-Coulter
CD158e1,e2	Z27.3.7	PE	Beckman-Coulter
CD336 (NKp44)	Z231	PE	Beckman-Coulter
CD337 (NKp30)	Z25	PE	Beckman-Coulter
CD335 (NKp46)	BAB281	PE	Beckman-Coulter
CD33	AC104.3E3	APC	Miltenyi Biotec
CD14	TÜK4	FITC	Miltenyi Biotec
HLA-DR	AC122	PerCP	Miltenyi Biotec
CD16	REA423	FITC	Miltenyi Biotec
CD11a (LFA-1)	TS2/4	PerCP	BioLegend
CD11a (LFA-1)	TS2/4	FITC	BioLegend
CD7	CD7-6B7	FITC	BioLegend
CD335 (NKp46)	9E2	eFluor450	BioLegend
CD117 (c-KIT)	104D2	PerCP-Cy5.5	BioLegend
CD161	HP-3G10	PerCP-Cy5.5	BioLegend
CD226 (DNAM-1)	11A8	PE	BioLegend
CD94	DX22	FITC	BioLegend
CD336 (NKp44)	P44-8	AF-647	BioLegend
CD337 (NKp30)	P30-15	AF-647	BioLegend
CD16	3G8	BV-421	BioLegend
CD127	A019D5	BV-421	BioLegend
CD132	TUGh4	APC	Biolegend
CD14	61D3	eFluor450	eBioscience-ThermoFisher
CD117	104D2	BV-421	eBioscience-ThermoFisher
CD14	61D3	APC-eFluor-780	eBioscience-ThermoFisher
IL-22	22URTI	PE	eBioscience-ThermoFisher
ROR-γt	AFKJS-9	PE	eBioscience-ThermoFisher
EOMES	WD1928	eFluor-660	eBioscience-ThermoFisher
Perforin	dG9	PE	eBioscience-ThermoFisher
IFN-γ	4S.B3	eFluor450	eBioscience-ThermoFisher
TNF-α	MAb11	eFluor450	eBioscience-ThermoFisher
CXCL8	6217	PE	R & D system
pSTAT3 XP rabbit (Tyr705)	D3A7	Unconjugated	Cell signaling
pSTAT5 XP rabbit (Tyr694)	D47E7	Unconjugated	Cell signaling
Goat-anti rabbit (IgG H + L) IgG1	A27034	AF-488	Invitrogen-ThermoFisher
CD122	Mik-β2	PE	BD Pharmingen

### Cell cytotoxicity assay

Cell cytotoxicity was analyzed in a 4 h 51Cr-release assay against human leukemic K562 cell line. Cells were counted, washed and plated. Effector/Target (E/T) cell ratio is 2/1. The effector target ratio was normalized to the numbers of CD56^+^ cells present in each culturing conditions. To this end, we adjusted the number of effector cells in each condition accordingly to the CD56^+^ cells present in the cultures and to the cell count performed simultaneously by using MACSQuant flowcytometer. Experiments were performed in duplicates. Data are expressed as percentage of target cell lysis.

### Intra-cytoplasmic cytokine, cytolytic granules, and TF expression assays

To detect cytokines, cells cultured in different conditions, were washed, suspended and over night stimulated with IL-12 (10 ng/ml), IL-15 (50 ng/ml), IL-18 (100 ng/ml), or IL-1β (50 ng/ml), IL-7 (50 ng/ml), IL-23 (50 ng/ml) (Peprotech, UK) in the presence of monensin (GolgiStop) or brefeldin (GolgiPlug) (Biosciences), respectively. For intra-cytoplasmic cytokine and cytolytic granules analyses, cells were stained for surface markers and then fixed and permeabilized with Fixation and Permeabilization Kit (BD Biosciences, New Jersey USA). Then, cells were incubated with cytokine- or Perforin- specific mAb. To detect TF expression, cells were suspended in 5% BSA buffer, stained for surface markers, subsequently fixed with Transcription Factor Staining Buffer Set (eBioscience-ThermoFisher) and stained for RORγt, and EOMES TF; instead, to detect expression of pSTAT3 and pSTAT5, cells were fixed with PFA 4% and methanol 100% and after that cells were stained with anti-pSTAT antibodies.

### Statistical analysis

Prism GraphPad software was used for statistical analysis. We considered significant *P* ≤ 0.05.

## Results

### TKI inhibit *in vitro* proliferation of myeloid and lymphoid precursors from CD34^+^ HSC

**Figure 1:**
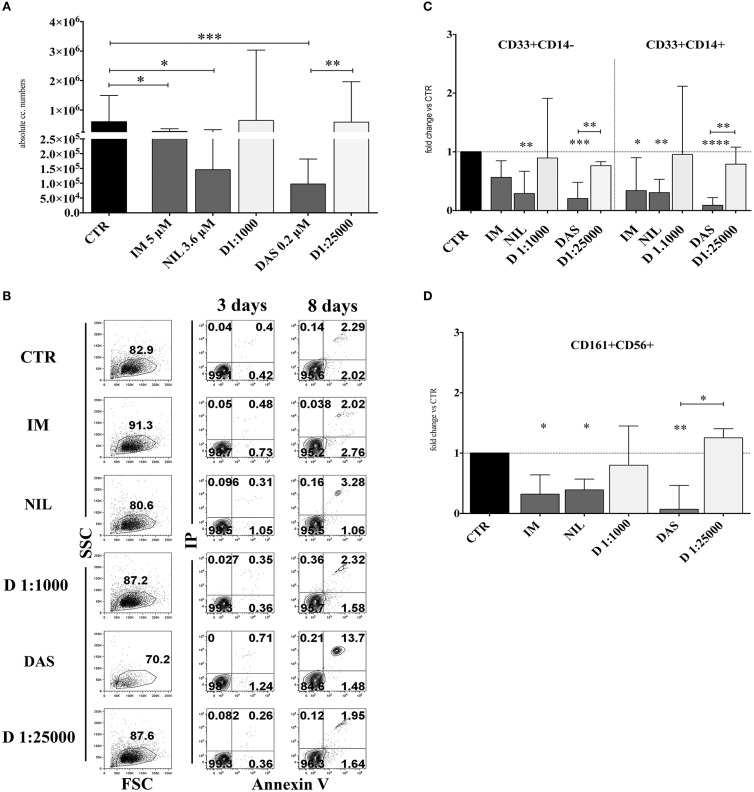
TKI inhibit the *in vitro* cell differentiation of CD34^+^HSC toward myeloid and lymphoid cells. UCB-CD34^+^ cells were purified and cultured with cytokine-mix medium in the absence (CTR, control) or in the presence of different TKI: Imatinib 5 μM (IM) Nilotinib 3,6 μM (NIL), Dasatinib 0,2 μM (DAS) at the plasma peak concentrations and DMSO at the corresponding concentrations of the drugs (D 1:1000 and D 1:25000). After 15 days of culture, cells were counted and analyzed by flow-cytometry for the indicated surface markers. **(A)** The histogram shows the absolute mononucleated cell number recovery in CTR or TKI cultures. The data are represented as the median with interquartile range obtained by ten independent experiments and analyzed by Kruskal-Wallis multiple comparison test (*p < 0.05;**p < 0.005;***p < 0.0005). **(B)** Dot plots show the morphological features and Annexin V/PI staining observed in the cell precursors undergoing *in vitro* NK cell differentiation in the presence of different culturing conditions: control (CTR), Imatinib 5 μM (IM), Nilotinib 3,6 μM (NIL), Dasatinib 0,2 μM (DAS) and DMSO (D 1:1000 and D 1:25000). The cells were undergone to immunofluorescence tests after 3 and 8 days of culture. **(C)** The histogram represents the fold change of absolute cell numbers of CD33^+^CD14^−^ and CD33^+^CD14^+^ cells recovered in TKI and DMSO cultures as compared to CTR, arbitrarily normalized to one. The data are expressed as the median values with interquartile range obtained by ten independent experiments. Data obtained in different culturing conditions were compared to CTR and analyzed by Wilcoxon signed-rank test (*p < 0.05;**p < 0.005;***p < 0. 0005;****p < 0.00005). Comparison between the different TKI culturing conditions and the different DMSO diluition culturing conditions was analyzed by Kruskal–Wallis multiple comparison test (*p < 0.05; **p < 0.005). **(D)** The histogram represents the fold change of CD161^+^CD56^+^ absolute cell number recovered in the presence of TKI and DMSO as compared to CTR, arbitrarily normalized to one. Data are represented as the median values with interquartile range obtained by ten independent experiments and comparison with CTR was analyzed by Wilcoxon signed-rank test (*p < 0.05;**p < 0.005). Comparison between the different TKI culturing conditions and the different DMSO dilution culturing conditions was analyzed by Kruskal–Wallis multiple comparison test (*p < 0.05).

In order to analyze the effects of different TKI on *in vitro* NK cell differentiation, UCB-CD34^+^ HSC were isolated and cultured with cytokines (SCF, FLT3-l, IL-7, IL-15 and IL-21, see section Material and Methods), suitable to promote NK cell differentiation, either in the absence (control = CTR) or in the presence of different TKI at plasmatic concentrations: Imatinib 5 μM (IM), Nilotinib 3.6 μM (NIL), Dasatinib 0.2 μM (DAS). DMSO was used as vehicle control. After 15 days of culture, cells were counted and analyzed for informative surface markers. As shown in Figure 1A, TKI led to a decreased mono-nucleated cell recovery that was particularly sharp in the case of Dasatinib. Evaluation of the surface staining for Annexin V after 3 and 8 days of culture suggested that the strong reduction of cell numbers detected in the presence of Dasatinib may be due to an increased programmed cell death, occurring during the first week of culture (Figure 1B). The analysis of informative cell surface markers indicated that the recovery of both CD33^+^CD14^−^ myeloid cells and CD33^+^CD14^+^ monocytes, was sharply reduced in the presence of all TKI analyzed (Figure 1C). Remarkably, all TKI induced a significant reduction of CD161^+^CD56^+^ absolute cell numbers as compared to controls. Also in this case, the effect was most striking in the presence of Dasatinib (Figure 1D). Of note, results obtained in cultures performed in the presence of Dasatinib displayed a sharp significant difference also with cultures performed in the presence of DMSO 1:25,000 (Figures 1A,C,D). Since cultures performed with DMSO at the final concentrations of 1:25,000 did not substantially differ in cell recovery from the CTR cultures, data on the cultures performed with DMSO at this concentration will not be shown any further.

### Dasatinib skews *in vitro* cell differentiation of CD161^+^CD56^+^ precursors toward ILC3

**Figure 2:**
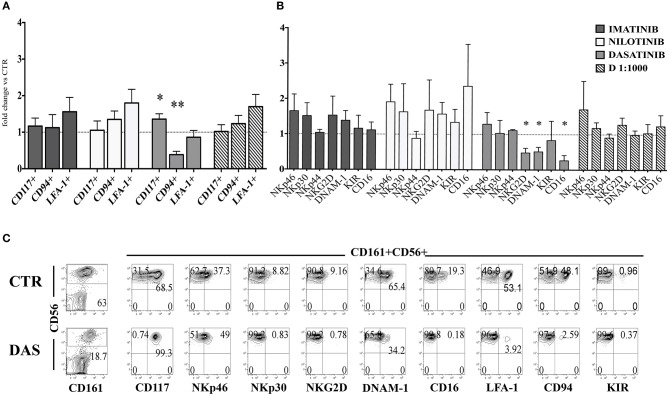
Dasatinib inhibits NK cell generation from CD34^+^ precursors. CD161^+^CD56^+^ cells developed in the absence (CTR) or in the presence of Imatinib 5 μM (IM), Nilotinib 3.6 μM (NIL), Dasatinib 0.2 μM (DAS) and DMSO 1:1000 (D 1:1000) were analyzed after 25 days of culture for the expression of the indicated surface markers. **(A)** The histogram represents the fold change of CD161^+^CD56^+^CD117^+^, CD161^+^CD56^+^CD94/NKG2A^+^, and CD161^+^CD56^+^LFA-1^+^ cell percentages, detected in the presence of the indicated TKI or DMSO, as compared to CTR cultures, arbitrarily normalized to one. Data are expressed as mean values ± SEM obtained by ten independent experiments and analyzed by Wilcoxon signed-rank test (*p < 0.05;**p < 0.005). **(B)** The histogram represents the fold change of CD161^+^CD56^+^ cell percentages expressing NKp46, NKp30, NKp44, NKG2D, DNAM-1, KIR, and CD16 receptors, developed in the presence of the indicated TKI or DMSO as compared to CTR cultures, arbitrarily normalized to one. Data are expressed as mean values ± SEM of ten independent experiments and analyzed by Wilcoxon signed-rank test (*p < 0.05). **(C)** Flow cytometric analyses show surface staining for the indicated surface markers expressed by CD161^+^CD56^+^ cells obtained after 25 days of culture in the absence (CTR) or Dasatinib 0.2 μM (DAS). Representative experiment out of ten.

**Figure 3:**
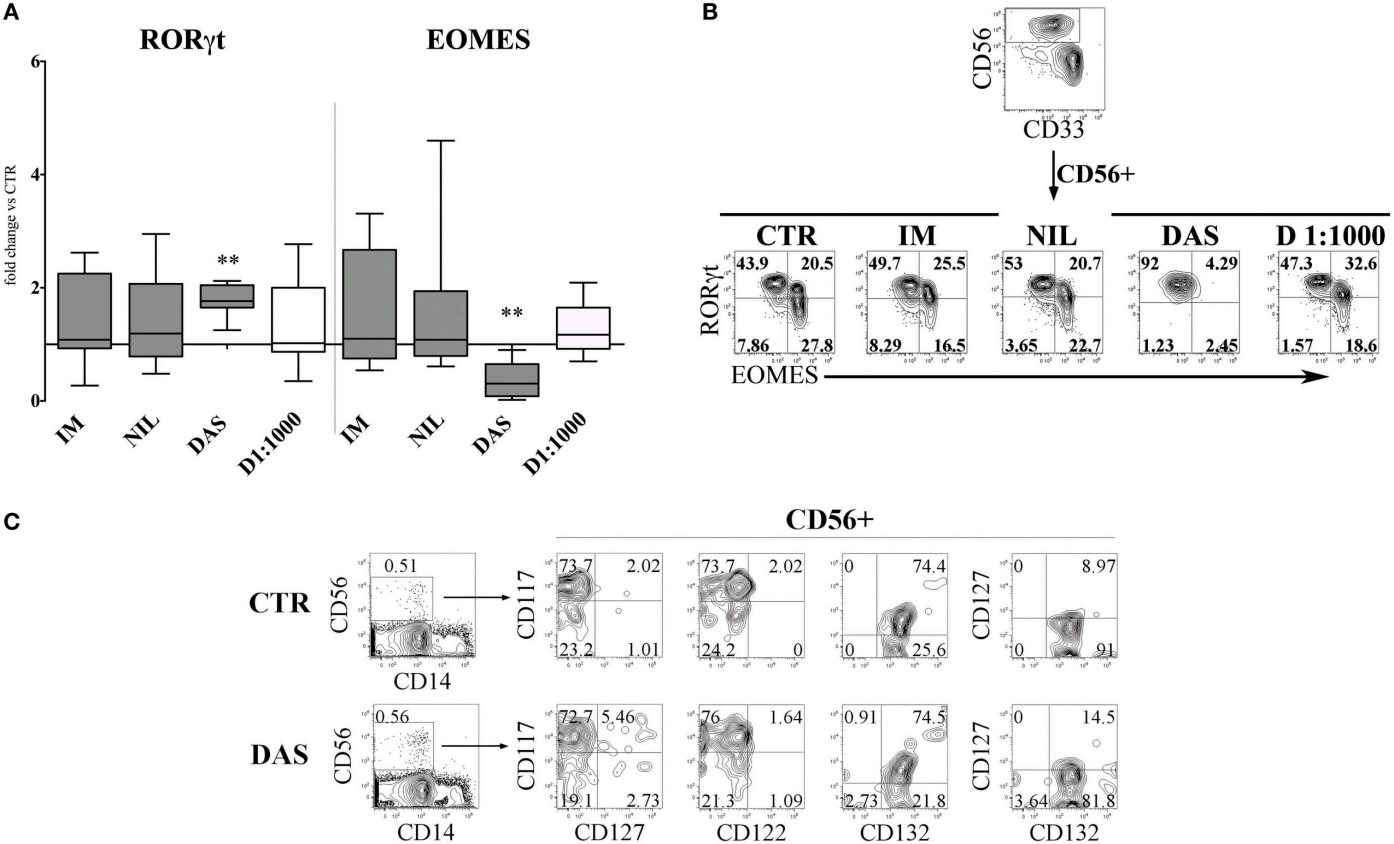
Dasatinib affects the CD56^+^ cell repertoire, favoring the enrichment of CD56^+^CD117^+^CD94/NKG2A^−^RORγt^+^ ILC3. CD161^+^CD56^+^ cells developed in the absence (CTR) or in the presence of Imatinib 5 μM (IM), Nilotinib 3.6 μM (NIL), Dasatinib 0.2 μM (DAS) and DMSO 1:1000 (D 1:1000) were analyzed by flow-cytometry after 25 days of culture for the expression of transcription factors (TF). **(A)** The histogram represents the fold change of CD56^+^RORγt^+^ and CD56^+^EOMES^+^ cell percentages, obtained in the presence of the indicated TKI or DMSO, as compared to CTR, arbitrarily normalized to one. Data are expressed as median values with interquartile range obtained by ten independent experiments and analyzed by Wilcoxon signed-rank test (**p < 0.005). **(B)** Dot plots show intra-nuclear staining of RORγt and EOMES after gating on CD33^−^CD161^+^CD56^+^ cells, developed in the absence (CTR) or in the presence of the indicated TKI or DMSO. Representative experiment out of ten performed. **(C)** UCB-CD34^+^ cells were purified and cultured with cytokine-mix medium in the absence (control=CTR) or in the presence of Dasatinib 200 nM. After 6 days of culture cells were analyzed by flow-cytometry for the indicated surface markers. Flow cytometric analysis show representative experiment out of two.

Next, we analyzed the surface phenotype of CD161^+^CD56^+^ cells obtained after 25 days under the culture conditions indicated above. As shown in Figure 2A, analysis of the receptor repertoire of CD56^+^ cells revealed a significant reduction of CD56^+^CD94/NKG2A^+^ cells in the presence of Dasatinib, while the percentages of CD56^+^CD117^+^ cells were significantly increased. Accordingly, also the percentages of NKG2D^+^, DNAM-1^+^, and CD16^+^ cells were reduced as compared to controls (Figure 2B). Figure 2C shows a representative experiment: in the presence of Dasatinib, a major decrease of percentages of CD56^+^ cells could be detected. Moreover, the majority of CD56^+^ cells were represented by CD117^+^LFA-1^−^CD94/NKG2A^−^CD16^−^ cells, a subset that may include both ILC3 and Stage III NK cell precursors (27). Thus, we further analyzed the expression of RORγt and Eomes TF, which allows discriminating ILC3 and stage IV/V NK cells. This analysis revealed a significant increase of CD56^+^ RORγt^+^ ILC3 and a significant decrease of CD56^+^ Eomes^+^ NK cells as compared to controls (Figures 3A,B), Of note, we could detect higher percentages of CD56^+^CD117^+^CD127^+^(CD132^+^) cells in cultures performed in the presence of Dasatinib as compared to controls, while CD122^+^ cells were virtually undetectable (Figure 3C).

### CD161^+^CD56^+^ cells developed in the presence of dasatinib express higher percentages of IFN-γ^+^ cells but display a reduced cytolytic activity

The altered composition of CD56^+^ cell subsets occurring in the presence of Dasatinib, suggests that this compound may affect NK cell differentiation and proliferation. To verify whether TKI could also affect the functional activity of CD56^+^ cells, we analyzed the intra-cytoplasmic cytokine expression and the cytolytic activity against the NK-susceptible K562 human leukemia cell line.

**Figure 4:**
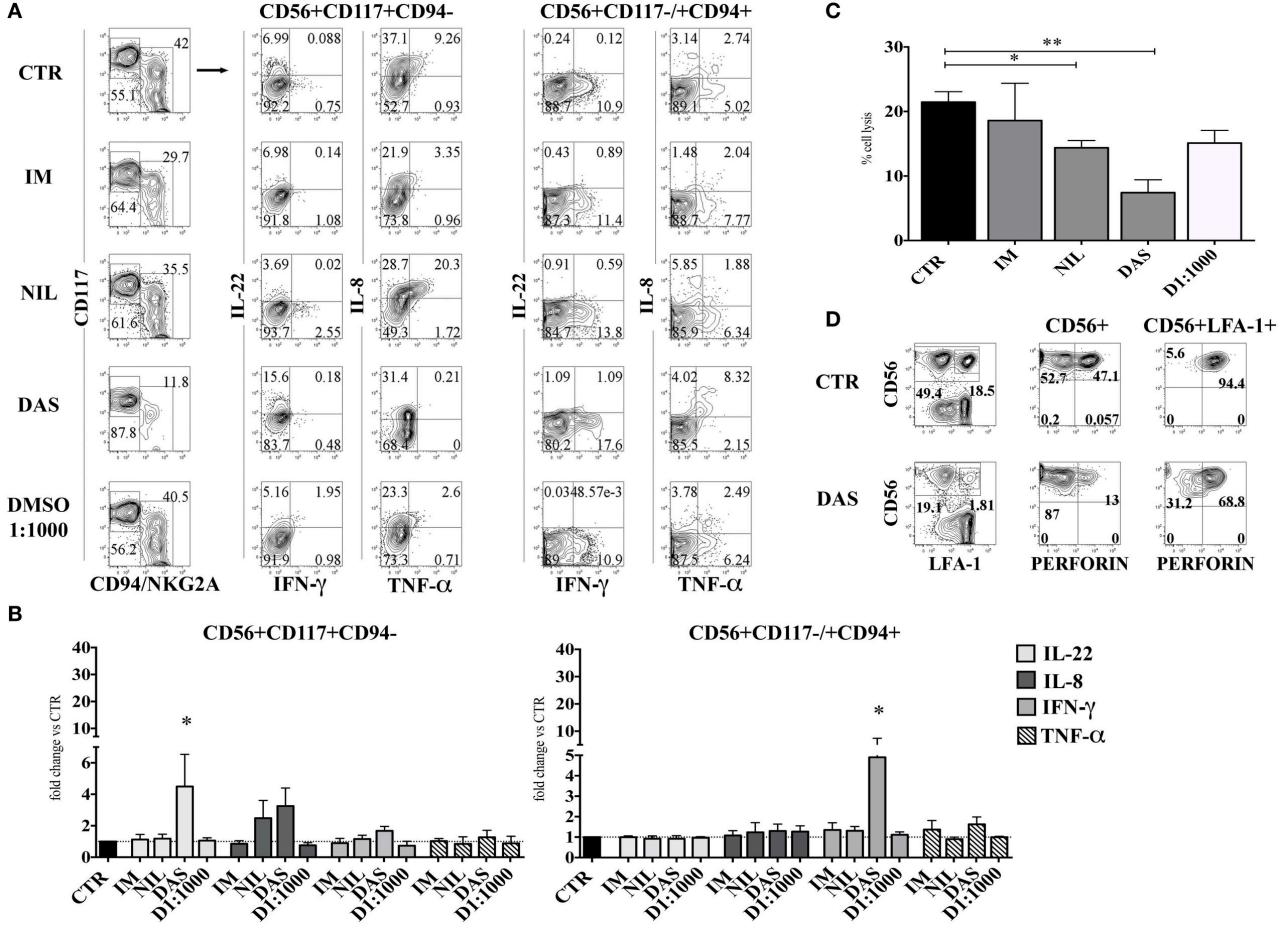
Dasatinib increases the percentages of IL-22-producing CD56^+^CD117^+^CD94^−^ and of IFN-γ-producing CD56^+^CD117^+/−^CD94^+^ cells while inhibits cytolytic activity of CD56^+^ cells. After 25 days CD56^+^cells developed in the presence of Imatinib 5 μM (IM), Nilotinib 3.6 μM (NIL), Dasatinib 0.2 μM (DAS) and DMSO 1:1000 (D 1:1000) were analyzed for the intra-cytoplasmic cytokine expression and for cell cytotoxicity. **(A,B)** CD56^+^ cells, developed in the absence or in the presence of different TKI or DMSO 1:1000 were o.n. stimulated with IL-1β+IL-7+IL-23, to induce the production of IL-22 and TNF-α and IL-8 or with IL-12+IL-15+IL-18, to favor the production of IFN-γ. Cells were analyzed for intra-cytoplasmic cytokine expression of IL-22, IL-8, IFN-γ, and TNF-α cytokines by flow cytometry. **(A)** Flow cytometry analysis show representative experiment out of seven: dot plots represent the intra-cytoplasmic cytokine staining in CD56^+^CD117^+^CD94/NKG2A^−^ and CD56^+^CD117^−/+^ CD94/NKG2A^+^ cell subsets. **(B)** The histograms represent the fold change of IL-22, IL-8, IFN-γ, and TNF-α positive cell percentages detected in CD56^+^CD117^+^CD94/NKG2A^−^ and CD56^+^CD117^−/+^CD94/NKG2A^+^ cell subsets upon appropriate cytokine stimulation, as compared to CTR, arbitrarily normalized to one. Data are expressed as mean percentage ± SEM of ten independent experiments and analyzed by Wilcoxon signed-rank test (*p < 0.05). **(C)** The histogram shows the percentage cell lysis of human leukemic line K562 by CD161^+^CD56^+^ cells. Effector/target (E/T) cell ratio was 2/1. Data are expressed as mean percentage ± SEM of four independent experiments and analyzed by Kruskal–Wallis multiple comparison test (*p < 0.05; **p < 0.005). **(D)** CD56^+^ cells were analyzed for intra-cytoplasmic expression of Perforin. Dot plots display Perforin intra-cytoplasmic staining in total CD56^+^ gated cells and in CD56^+^LFA-1^+^(CD94^+^) gated cells. Representative experiment out of four performed.

CD56^+^ lymphoid cells, developed in the absence or in the presence of different TKI, were stimulated either with IL-1β, IL-7, and IL-23, or with IL-12, IL-15, and IL-18 and analyzed for intra-cytoplasmic cytokine expression by flow cytometry. Figures 4A,B shows that CD56^+^CD117^+^CD94^−^ ILC3 subset, generated in the presence of Dasatinib, display higher percentages of IL-22^+^ and IL-8^+^ cells and a slight increase of IFN-γ^+^ cells. Interestingly, in the presence of Dasatinib, also the small CD56^+^CD117^−/+^CD94^+^ cell subset, expressed higher proportions of IFN-γ^+^ cells as compared to the other culturing conditions (Figures 4A,B). Analysis of the cytolytic activity against K562 target cells, indicated that CD56^+^ cells generated in the presence of Nilotinib or Dasatinib, were significantly less cytolytic (Figure 4C). Of note, the decreased cytotoxicity, observed in the presence of Dasatinib, was associated to lower percentages of CD56^+^ Perforin^+^ cells as compared to controls (Figure 4D).

### Inhibition of src kinases skews CD56^+^ cell repertoire undergoing *in vitro* cell differentiation

**Figure 5:**
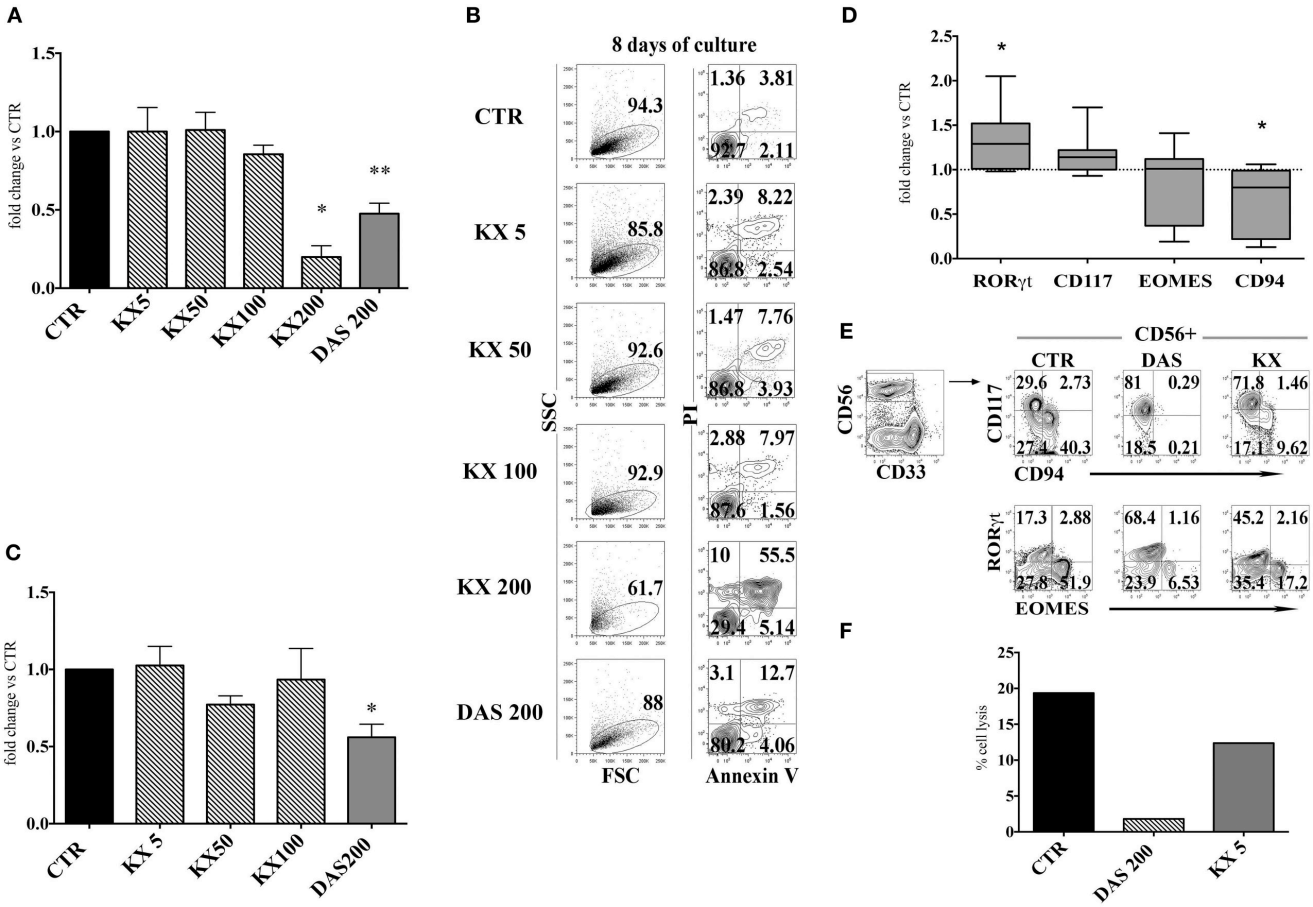
KX2-391 Src kinases inhibitor favors the development of CD56^+^CD117^+^RORγt^+^ cells, while inhibits generation and cytotoxicity of CD56^+^CD94/NKG2A^+^EOMES^+^ cells. UCB-CD34^+^ cells were purified and cultured with cytokine-mix medium in the absence (control=CTR) or in the presence of KX2-391 src kinase-inhibitor at different concentrations (5, 50, 100 nM) or Dasatinib at the plasma peak concentration (200 nM). **(A)** The histogram represents the absolute cell numbers obtained after 15 days of culture, in the presence of KX2-391 inhibitor at different concentrations and Dasatinib, as compared to control, arbitrarily normalized to one. The data are represented as mean±SEM obtained by nine independent experiments and analyzed by Wilcoxon signed-rank test (*p < 0.05; **p < 0.005). **(B)** Dot plots show the morphological features and Annexin V/PI staining observed in the cell precursors undergoing *in vitro* NK cell differentiation in the presence of different culturing conditions: control (CTR), KX2-391 at different concentrations and Dasatinib. The cells were undergone to immunofluorescence tests after 8 days of culture. **(C)** The histogram shows the fold change of CD161^+^CD56^+^ absolute cell number recovered after 15 days of culture in the presence of KX2-391 inhibitor at different concentrations and Dasatinib, as compared to control, arbitrarily normalized to one. The data are represented as mean ±SEM obtained by nine independent experiments and analyzed by Wilcoxon signed-rank test (*p < 0.05). **(D)** Box and Whiskers plots represent the fold change of CD56^+^RORγt^+^, CD56^+^CD117^+^, CD56^+^EOMES^+^ and CD56^+^CD94/NKG2A^+^ cells, obtained after 25 days of culture in presence of KX2-391 5nM, as compared to control. The data are obtained by seven independent experiments and analyzed by Wilcoxon signed-rank test (*p < 0.05). **(E)** Dot plots show analysis of CD94/NKG2A, RORγt and EOMES TF in CD56^+^ cells, in the absence (= CTR) or in the presence of KX2-391 5 nM or Dasatinib 200 nM. Representative experiment out of three. **(F)** The histogram represents the percentage cell lysis of human leukemic line K562 by CD161^+^CD56^+^ cells, after 27 days of culture, in the presence of KX2-391 5 nM and Dasatinib 200 nM, as compared to control. Representative experiment out of two performed.

It has been reported that Dasatinib, but not Imatinib and Nilotinib, exerts an inhibitory effect on the family of Src kinases: thus, it is possible that the lower numbers of CD56^+^ cells, detected in the presence of this compound, may reflect an inhibitory effect on Src kinases occurring at the level of cell precursors (36, 39). To test this possibility, CD34^+^HSC were cultured in appropriate cytokine mix medium in the absence or in the presence of different concentrations of KX2-391 (5, 50, 100, and 200 nM) a non-selective Src kinase inhibitor, or in the presence of Dasatinib (200 nM). After 15 days of culture, cells were counted. KX2-391 did not significantly impair cell proliferation at different drug concentrations with the exception of highest dose (200 nM), in which an extensive cell death could be detected (Figures 5A,B). Moreover, there were no significant variations in CD56^+^ cell percentages in the presence of lower concentrations of KX2-391 inhibitor as compared to controls (Figure 5C). However, analysis of the cell surface phenotype revealed that KX2-391 could induce substantial modifications of the subset composition within the CD56^+^ cell population, similar to those detected in the presence of Dasatinib. Thus, as shown in Figures 5D,E, even in the presence of the lowest concentration of KX2-391 (i.e., 5 nM), there was an increased expression of RORγt TF and of CD117, paralleled by the reduced expression of Eomes TF and of CD94/NKG2A. Accordingly, CD56^+^ cells undergoing differentiation in the presence of Dasatinib 200 nM or KX2-391 5 nM, displayed a lower cytolytic activity against K562 target cells as compared to control (Figure 5F).

**Figure 6:**
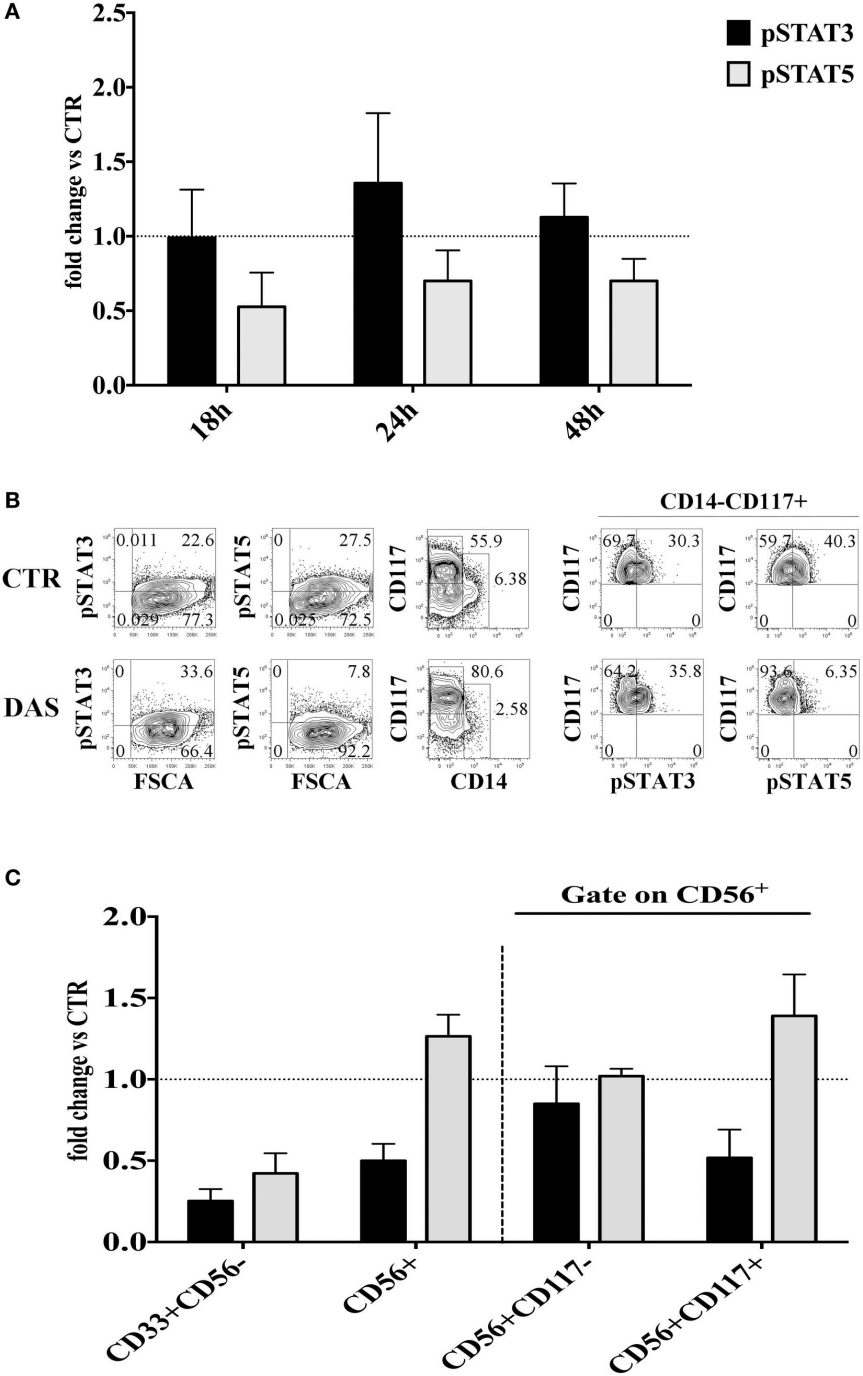
Analysis of the Dasatinib-mediated effect on STAT3 and STAT5 phosphorylation in CD34^+^ cells undergoing differentiation toward CD56^+^cells at different culture time intervals. **(A)** UCB-CD34^+^ cells were isolated and cultured with cytokine-mix medium, in the absence or in the presence of Dasatinib 200 nM for 18, 24, and 48 h. Cells were analyzed for the expression of pSTAT3 and pSTAT5 signaling proteins. The histograms show the fold changes of CD34^+^pSTAT3^+^ and CD34^+^pSTAT5^+^ positive cell percentages detected in the presence of Dasatinib, as compared to control, arbitrarily normalized to one. Data are expressed as mean values ± SEM obtained by four independent experiments. **(B)** UCB-CD34^+^ cells were isolated and cultured with cytokine-mix medium, in the absence or in the presence of Dasatinib 200 nM for 72 h. Flow cytometry analyses show the intra-cytoplasmic expression of pSTAT3 and pSTAT5 on total cell population and on CD14^−^CD117^+^ gated cells. **(C)** CD56^+^ cells obtained from UCB-CD34^+^ cells after 20 days of culture, in the absence (CTR) or in the presence of Dasatinib 200 nM were analyzed for the expression of pSTAT3 and pSTAT5 signaling proteins. The histogram shows the fold change of CD56^+^pSTAT3^+^ and CD56^+^pSTAT5^+^ cell percentages detected in the presence of Dasatinib, as compared to control, arbitrarily normalized to one. Data are expressed as mean values ± SEM obtained by four independent experiments.

It has been shown that Dasatinib can inhibit the Signal Transducer and Activator of Transcription (STAT) 3 and STAT 5 protein phosphorylation through the inhibition of Src kinases. Thus, the Dasatinib–mediated effects could also be due to the inhibition of STAT3/STAT5 signaling pathways. To address this issue we investigated whether Dasatinib could inhibit STAT3/STAT5 phosphorylation in CD34^+^ cell precursors since early days of *in vitro* culture. Thus, CD34^+^ cells were cultured with cytokine mix medium in the absence or in the presence of Dasatinib, and STATs protein phosphorylation was analyzed at different time intervals, i.e., 18/24/48 h of culture. A reduction of pSTAT5^+^ cell percentages was detected while the percentages of pSTAT3^+^ cells were similar or higher than those detected in control cultures at all the time intervals analyzed (Figure 6A). Of note, after 72 h of culture in the presence of Dasatinib, we could still observe lower pSTAT5^+^ cell percentages as compared to control, and increase of CD117^+^ cell percentages (Figure 6B). On the other hand, analysis performed at 20 days of culture, showed that CD56^+^ cell subsets developed in the presence of Dasatinib, contained higher percentages of pSTAT5^+^ cells as compared to controls, while myeloid cells displayed a marked reduction of both pSTAT3^+^ and pSTAT5^+^ cells (Figure 6C).

## Discussion

In this study we analyzed the effect of the TKI inhibitors Imatinib, Nilotinib, and Dasatinib on NK cell differentiation from UCB-derived CD34^+^ cell precursors. We show a sharp inhibition of cell proliferation and a reduced recovery of both myeloid and CD161^+^CD56^+^ lymphoid cells. More importantly, Dasatinib skewed the subset composition of CD161^+^CD56^+^ cell population favoring the generation of RORγt^+^IL-22^+^ ILC3 and inhibiting both proliferation and function of cytotoxic NK cells. Experiments using the KX2-391 Src-family kinase inhibitor suggest that the impairment of NK cell generation and function may be consequent to the inhibitory effect mediated by Dasatinib on Src-family kinases and the early impairment of STAT5 signaling in CD34^+^ cell precursors.

The TKI therapy in CML or Ph^+^ALL patients has dramatically improved the prognosis of these patients. However, the development of resistance and leukemia relapse, in particular upon therapy discontinuation, still represents a major problem. Thus, in recent years, a major research focus has been to improve both DMR and TFR (8). Notably, a positive clinical outcome in these patients appears to correlate with an efficient immune response allowing the control of the CML residual disease (13). Since NK cells are thought to play a relevant role in this process, it was important to clarify whether TKI could influence not only the repertoire and function of circulating mature NK cells, but also their development from CD34^+^ HSC. To this end, we took advantage of an *in vitro* model of NK cell differentiation from CD34^+^ cell precursors that allows the generation of different ILC subsets. In addition, it provides a useful tool to analyze factors/drugs that may modify such process (27, 40). In the present experiments, CD34^+^ cells were cultured in the presence of Imatinib, Nilotinib, and Dasatinib at plasmatic peak concentrations, to reproduce drug concentrations present in PB and BM of TKI-treated patients. An inhibitory effect of TKI on precursor cell proliferation could be expected, since these compounds are known to interfere with the SCF/c-KIT (CD117) transduction pathway that plays a key role in the early steps of CD34^+^ cell activation and proliferation (37, 41). However, the impairment of cell proliferation and recovery was associated with an increase of programmed cell death only in Dasatinib culture condition, in which apoptosis was clearly detectable during the first week of culture. Although all TKI analyzed induced a decrease of CD161^+^CD56^+^ cell recovery, Dasatinib exerted a more marked inhibitory effect. Moreover, only Dasatinib significantly skewed the CD161^+^CD56^+^ cell repertoire favoring CD117^+^CD94^−^LFA-1^−^-RORγt^+^ ILC3, while CD117^−^CD94^+^LFA-1^+^Eomes^+^ NK cells were sharply reduced. The effect of Dasatinib on ILC commitment was detectable at early stages, since analysis performed at day 6 of culture revealed an increase of CD117^+^CD127^+^CD132^+^ cells, representing ILC precursors, while CD122 expression was undetectable. Previous studies on factors that may influence human ILC *in vitro* development, have shown that the presence of SCF is required to favor the *in vitro* differentiation of ILC3 in the presence of IL-7 or IL-15, while IL-15 and IL-7, alone or in combination with other pro-inflammatory cytokines, skew precursor cell differentiation toward NK cells (40, 42).

The SCF/c-kit transduction pathway involves STAT3 protein phosphorylation, IL-15 pathway preferentially uses STAT5 signaling protein, while IL-7 can exploit both (37, 43). It has been shown that Dasatinib inhibits the STAT3 and STAT5 signaling pathways through the inhibition of Src kinases, leading to a durable inhibition of STAT5 phosphorylation, but only a transient inhibition of STAT3 phosphorylation in CD34^+^ cells isolated from CML patients at diagnosis (39). Our analysis of STAT3/STAT5 phosphorylation in CD34^+^HSC revealed a higher and durable reduction of pSTAT5^+^ cells as compared to pSTAT3^+^ cells upon cell culture with Dasatinib during first days of culture. Thus, it is conceivable that the early expression of CD117 and CD127 on Dasatinib-treated cells may favor the SCF/CD117- and IL-7/CD127–mediated STAT3 transduction triggering pathway, thus favoring ILC precursors proliferation and survival.

**Figure 7:**
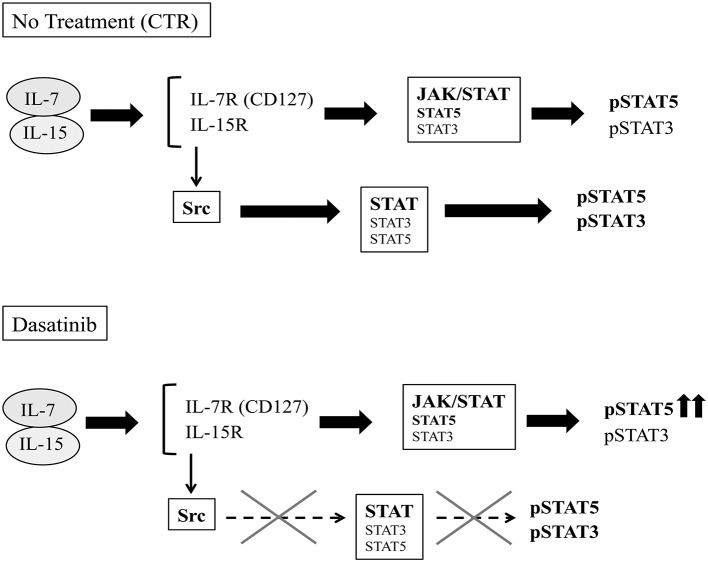
Schematic representation of potential effects of Dasatinib on IL-7/IL-15 transduction pathways. IL-7 and IL-15 cytokines can signal both through the JAK-STAT and the Src pathways.After 20 days of culture, in the control condition, the cytokine receptors engagement by IL-7 and IL-15 leads both to JAK- and Src- STAT-mediated recruitment and phosphorylation. In the presence of Dasatinib, Src-mediated STAT phosphorylation pathway is abrogated, while cytokine-driven JAK-activation pathway would provide compensatory signals, allowing STAT5 phosphorylation.

Functional analyses indicate that Dasatinib does not affect cytokine production by ILC3 but rather induces increases of the percentages of CD56^+^ CD117^+^RORγt^+^IL-22 producing cells. Interestingly, the few NK cells, undergoing differentiation in the presence of Dasatinib, contained higher percentages of IFN-γ^+^cells. We also detected a slight increase of IL-8^+^/IFN-γ^+^ILC3 undergoing differentiation in the presence of Dasatinib. It has been reported that lymphocyte mobilization in Dasatinib-treated patients is frequently associated with adverse effects such as pleural effusion, autoimmune-like syndromes and colitis (29). Of note, ILC3 are thought to play a relevant role in intestinal inflammation (25). In addition, they may exacerbate intestinal inflammation due to their ability to differentiate toward IFN-γ-producing ILC1 cells upon IL-12 stimulation (44). Thus, the inflammatory side effects induced by Dasatinib in mucosal tissues may reflect, at least in part, increases in the production of IL-22, IL-8, and IFN-γ by ILC. Of note, the increase of IFN-γ-expressing CD56^+^ cells detectable after 25 days of culture in the presence of Dasatinib, paralleled the increase of CD56^+^pSTAT5^+^cell percentages. These data may suggest that a prolonged exposure to IL-7 and IL-15 may provide compensatory signals, allowing lymphoid cell cytokine production even in the presence of a chronic exposure to Dasatinib (45) (Figure 7).

The cytolytic activity of CD56^+^ NK cells developed in the presence of Dasatinib was impaired. It is conceivable that the reduction of cytotoxicity may reflect the lower number of mature NK cells however, we could also detect a decrease of Perforin content in the few mature NK cells present in Dasatinib cultures. In addition, it should be considered the inhibitory effect exerted by Dasatinib on Src kinases and on ERK protein phosphorylation (p-ERK), required for the cytolytic degranulation process (39, 46, 47). Indeed, our experiments using KX2-391 inhibitor would confirm that the effect on the CD56^+^ cell repertoire induced by Dasatinib, may be due to the impairment of Src kinase pathway. Notably, KX2-391 induced massive cell apoptosis at the concentration of 200 nM while at lower concentrations the proportions of Annexin V^+^ cells were low. However, the use of KX2-391 at lower concentrations skewed the CD56^+^ cell repertoire toward CD56^+^CD117^+^CD94/NKG2A^−^ RORγt^+^ ILC3, similar to what detected in cultures performed in the presence of Dasatinib.

Studies in mice models have suggested that IL-15/STAT5 pathway may represent a central node in NK cell homeostasis and in the TF network that instructs ILC development (48–51). Our data indicate that exposure to Src kinase inhibitors may play a relevant a role in the inhibition of human NK cell development and in the acquisition of cytolytic activity, while the cytokine-driven STAT3 and STAT5 activation pathway may overcome Dasatinib-mediated inhibition and favor the preferential survival of CD117^+^ CD127^+^ ILC3 precursors and the subsequent cytokine production by both mature ILC3 and NK cells detectable at later time culture intervals.

It has been reported that Dasatinib, may induce cytotoxic CD56^+^CD57^+^ NK cell mobilization in the PB and BM of TKI-treated patients (29–31, 52). It is conceivable that different effects detectable *in vitro* vs. *in vivo* of Dasatinib-treatment may be consequent to the short half-life of the drug in plasma vs. its stable levels in cell cultures (53). Notably, it has been suggested that the incidence of lymphocytosis does not correlate with DMR, while the numbers and percentages of circulating cytotoxic NK cells and CTL were significantly higher in patients with DMR as compared to those with no DMR (30). The high degree of heterogeneity of cases analyzed may contribute to the variability of responses: patients may undergo different TKI administration protocols, and the immune system status (immunosuppression, viral reactivation, therapy-related BM/ lymphoid organs exhaustion) may greatly influence immune cell repertoire and function, having an impact on the clinical outcome of CML-treated patients (13).

In conclusion, our results suggest that Dasatinib may affect NK cell development, and offer new clues to better understand the signaling pathways that regulate development and proliferation of precursors toward NK cells and other ILC. The Dasatinib-induced skewing of ILC differentiation toward ILC3 and increasing of IFN-γ producing cells should be considered in patients with severe therapy-induced side effects or with limited responses to therapy. Moreover, in Dasatinib-treated patients, it will be important to better characterize both the lymphoid cell precursors and the mature cytolytic effector cells present in patients BM, i.e., the site where drug-resistant leukemic clones primarily reside (54). Finally, our study, could offer a clue for identifying new tools to design individualized timing and dosing of Dasatinib administration in order to obtain optimal responses without compromising the NK cell-based immunotherapeutic intervention.

## Ethics statement

The ethics committe that approved the study was Comitato Etico Regionale Liguria- Ospedale San Martino. Samples were from Liguria Cord Blood Bank. This study was carried out in accordance with the recommendations of UE guidelines for Good Clinical Practice and Comitato Etico Regionale Liguria- Ospedale San Martino with written informed consent from all subjects. All subjects gave written informed consent in accordance with the Declaration of Helsinki. The protocol was approved by the Comitato Etico Regionale Liguria- Ospedale San Martino.

## Author contributions

LD designed the experimental plan and performed experiments. EM designed the first set of experiments. LM analyzed results. CV designed the experimental plan, analyzed results and wrote the paper. MCM analyzed results. All the authors contributed to the critical review of the manuscript. CV and MM share senior authorship.

### Conflict of interest statement

The authors declare that the research was conducted in the absence of any commercial or financial relationships that could be construed as a potential conflict of interest.
